# Identification of Tumorigenic and Prognostic Biomarkers in Colorectal Cancer Based on microRNA Expression Profiles

**DOI:** 10.1155/2020/7136049

**Published:** 2020-08-03

**Authors:** Yuntao Shi, Yingying Zhuang, Jialing Zhang, Mengxue Chen, Shangnong Wu

**Affiliations:** ^1^Department of Gastroenterology, The Affiliated Huai'an No.1 People's Hospital of Nanjing Medical University, No. 1, Huanghe West Road, Huai'an 223300, China; ^2^Department of Medical Imaging, The Affiliated Huai'an No.1 People's Hospital of Nanjing Medical University, No. 1, Huanghe West Road, Huai'an 223300, China

## Abstract

**Objective:**

Although noncoding RNAs, especially the microRNAs, have been found to play key roles in CRC development in intestinal tissue, the specific mechanism of these microRNAs has not been fully understood.

**Methods:**

GEO and TCGA database were used to explore the microRNA expression profiles of normal mucosa, adenoma, and carcinoma. And the differential expression genes were selected. Computationally, we built the SVM model and multivariable Cox regression model to evaluate the performance of tumorigenic microRNAs in discriminating the adenomas from normal tissues and risk prediction.

**Results:**

In this study, we identified 20 miRNA biomarkers dysregulated in the colon adenomas. The functional enrichment analysis showed that MAPK activity and MAPK cascade were highly enriched by these tumorigenic microRNAs. We also investigated the target genes of the tumorigenic microRNAs. Eleven genes, including PIGF, TPI1, KLF4, RARS, PCBP2, EIF5A, HK2, RAVER2, HMGN1, MAPK6, and NDUFA2, were identified to be frequently targeted by the tumorigenic microRNAs. The high AUC value and distinct overall survival rates between the two risk groups suggested that these tumorigenic microRNAs had the potential of diagnostic and prognostic value in CRC.

**Conclusions:**

The present study revealed possible mechanisms and pathways that may contribute to tumorigenesis of CRC, which could not only be used as CRC early detection biomarkers, but also be useful for tumorigenesis mechanism studies.

## 1. Introduction

Colorectal cancer (CRC) has a high incidence in malignant tumors globally and a high mortality rate, making it the world's fourth most deadly cancer (after lung, liver, and stomach cancer) [1]. This situation is the same in China. According to the 2015 cancer statistics released by the National Cancer Center of China, the number of cases of colorectal cancer in 2015 was 376,300, and the prognosis of colon cancer patients was poor. In recent years, the mortality rate is on the rise. In 2015, the number of deaths due to colorectal cancer in China was 191,000, and the mortality rate was as high as 50.78% [2].

The genesis of colorectal cancer is the result of the interaction between genetic and environmental factors, and is a multigene, multistep process. Other risk factors include a family history of CRC, a diet low in fibers and folate and high in fat and red meat, alcohol, cigarette smoking, sedentary occupation, obesity, and diabetes [3]. It has been reported that the initial carcinogenic event attacks stem cells located at the bottom of the intestinal crypt [4]. CRC tumorigenesis can be driven by specific molecular alterations with functional effects in prosurvival signaling pathways such as p53 pathway, Wnt-*β*-catenin pathway, EGFR- (epidermal growth factor receptor-) MAPK (mitogen-activated protein kinase) pathway, PI3K (phosphatidylinositol 3-kinase) pathway, nuclear factor-kappa B (NF-*κ*B) pathway, and activator protein 1 (AP-1) pathway [5].

microRNAs (miRNAs) are small single-stranded noncoding RNA molecules about 22 nucleotides long and can posttranscriptionally regulate target genes' expression by binding to the 3′ untranslated region of mRNAs [6, 7]. They have important roles in diverse cellular processes including cell proliferation, differentiation, and apoptosis [8]. It is an important factor for tumorigenesis of colorectal cancer (CRC), and a potential biomarker for diagnosis, prognosis, and therapy of CRC [9]. To date, a lot of miRNAs have been implicated in CRC. For instance, miR-92a is a potential diagnostic biomarker for early detection of CRC, overexpressed in CRC, known to be involved in cell proliferation and apoptosis [10]. MiR-7 is a novel miRNA in CRC with reduced cell proliferation, increased apoptosis, and activated early cell cycle checkpoints for tumor suppressor function in vitro and reduced tumor growth in nude mouse xenografts [11]. In this study, we analyzed the microRNA expression profiles of 649 normal mucosa and 103 intestinal adenoma samples and aimed at finding some key tumorigenic microRNAs and their underlying mechanism and clinical significance, which might be useful for the tumorigenesis-related researches in colon cancer.

## 2. Methods

### 2.1. The microRNA Expression Profiles of Normal Mucosa, Adenoma, and Carcinoma

We downloaded the microRNA expression profiles of 649 normal mucosa samples, 103 intestinal adenoma samples from the GEO (Gene Expression Omnibus) database with accession number of GSE115513 [12]. The expression levels of 2,006 microRNAs were measured with Agilent Human miRNA V19.0 Microarray (https://www.ncbi.nlm.nih.gov/geo/query/acc.cgi?acc=GSE115513). The normalized data provided by Slattery et al. [12] was used to identify the differentially expressed microRNAs between normal mucosa and adenoma. Moreover, the microRNA expression profiles of The Cancer Genome Atlas (TCGA) colon adenocarcinoma (COAD) cohort were collected from the UCSC Xena database [13] (https://xena.ucsc.edu/) for further analysis.

### 2.2. Differential Expression Analysis

Wilcoxon rank-sum test and fold change were used to evaluate the significance of the microRNAs differentially expressed between the normal tissues and colon adenomas. The *P* values were adjusted by the false discovery rate (FDR) method. The microRNAs were deemed as differentially expressed if the FDR < 0.05 and fold change > 4 or <1/4.

### 2.3. Gene Set Overrepresentation Analysis of the microRNAs

The gene set overrepresentation analysis of the microRNAs was implemented by the online tool miEAA [14] to identify the gene ontology (GO) terms, KEGG pathways, and miRNA target genes enriched by the tumorigenic microRNAs.

### 2.4. Integrative Analysis of CNA and microRNA Expression in TCGA COAD Cohort

The genomic loci that the microRNAs were originated from were collected from the miRBase database. CNA datasets were downloaded from the UCSC Xena database [13] (https://xena.ucsc.edu/). The copy number statuses of the microRNAs were determined as amplifications or deletions if the log2 ratio > 0.6 or <-0.6. Wilcoxon rank-sum test was used to test the difference between the samples with and without CNAs.

### 2.5. Cox Regression Model-Based Survival Analysis

The survival analysis based on the Cox-regression model was implemented in R with package survival. The stepwise method was used for feature and model selection. Kaplan-Meier curve was used to visualize the survival probability for each group.

### 2.6. Support Vector Machine (SVM)

The SVM was trained based on the tumorigenic microRNAs using the R e1071 package. The Receiver Operating Characteristic (ROC) curve and area under the curve (AUC) value were used to evaluate the performance of the microRNAs in discriminating the CRC from normal tissues.

## 3. Results

### 3.1. The Tumorigenic microRNAs in Colorectal Cancer

**Figure 1:**
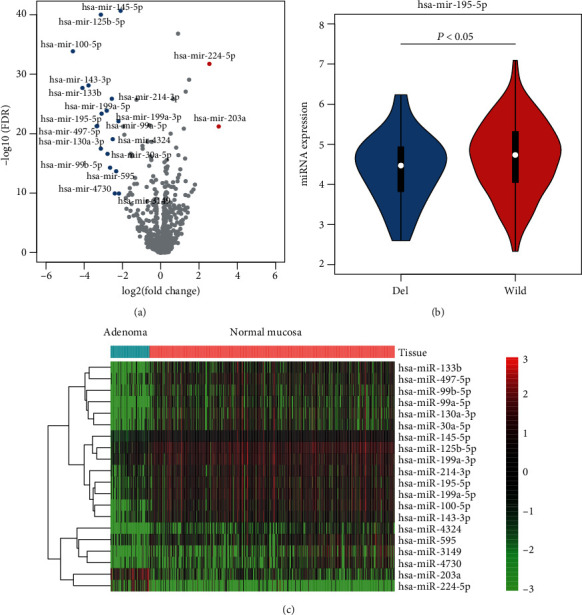
The tumorigenic microRNAs identified by differential expression analysis. (a) The upregulated and downregulated microRNAs were colored by red and blue, respectively. (b) The expression patterns of hsa-mir-195-5p in samples with and without CNAs in hsa-mir-195-5p. (c) The expression profiles of the tumorigenic microRNAs in adenomas and normal tissues.

To identify the tumorigenic microRNAs in colorectal cancer, we first collected the miRNA expression profiles of 103 colon adenomas and 649 normal mucosae. The differential expression analysis was conducted to identify the microRNAs that dysregulated in colon adenoma, which might be the critical regulators in the tumorigenesis of colorectal cancer. With stringent thresholds of FDR < 0.05 and fold change > 4 or <1/4, we identified two upregulated and 18 downregulated microRNAs (Figure 1(a)). Notably, the mir-224-5p and mir-203a were the two upregulated microRNAs in colon adenoma, which might act as oncogenic roles in colorectal cancer. Moreover, mir-195-5p, a well-recognized tumor suppressor in cancers, was observed to be downregulated in colon adenoma. Further analysis, the copy number status of this microRNA in TCGA colon adenocarcinoma (COAD) revealed that its downregulation was closely associated with its deletion in COAD patients (Figure 1(b)), suggesting that the downregulation may be caused by the genomic deletions. Furthermore, as shown in Figure 1(c), these tumorigenic microRNAs exhibited distinct expression patterns between the adenoma and normal mucosa.

### 3.2. Gene Ontology Terms and Pathways Enriched by the Tumorigenic microRNAs

**Figure 2:**
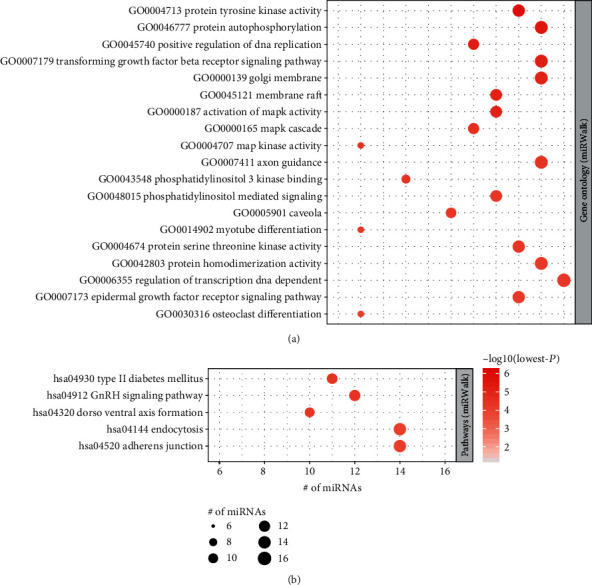
The functional enrichment analysis of tumorigenic microRNAs. Gene Ontology (GO) terms (a) and Kyoto Encyclopedia of Genes and Genomes (KEGG) pathways (b) were significantly enriched by the microRNAs (*P* < 0.0001).

We conducted overrepresentation enrichment analysis (ORA) of the 20 tumorigenic microRNAs using an online tool called miEAA [14]. We totally identified 19 gene ontology (GO) terms and 5 Kyoto Encyclopedia of Genes and Genomes (KEGG) pathways significantly enriched by these tumorigenic microRNAs (Figure 2). We observed that kinase-related GO terms, including protein tyrosine kinase activity, activation of MAPK activity, MAPK cascade, phosphatidylinositol 3 kinase binding, phosphatidylinositol mediated signaling, and protein serine-threonine kinase activity, were frequently enriched by these tumorigenic microRNAs, suggesting that the dysregulation of these tumorigenic microRNAs might cause altered kinase activities, thereby promoting the formation of adenoma (Figure 2(a)). The KEGG pathways were mainly enriched in the interactions between cell and extracellular matrix, such as endocytosis and adherens junction (Figure 2(b)), indicating that these interactions had been changed in adenoma.

### 3.3. Prediction of Tumorigenic microRNA Target Genes

**Figure 3:**
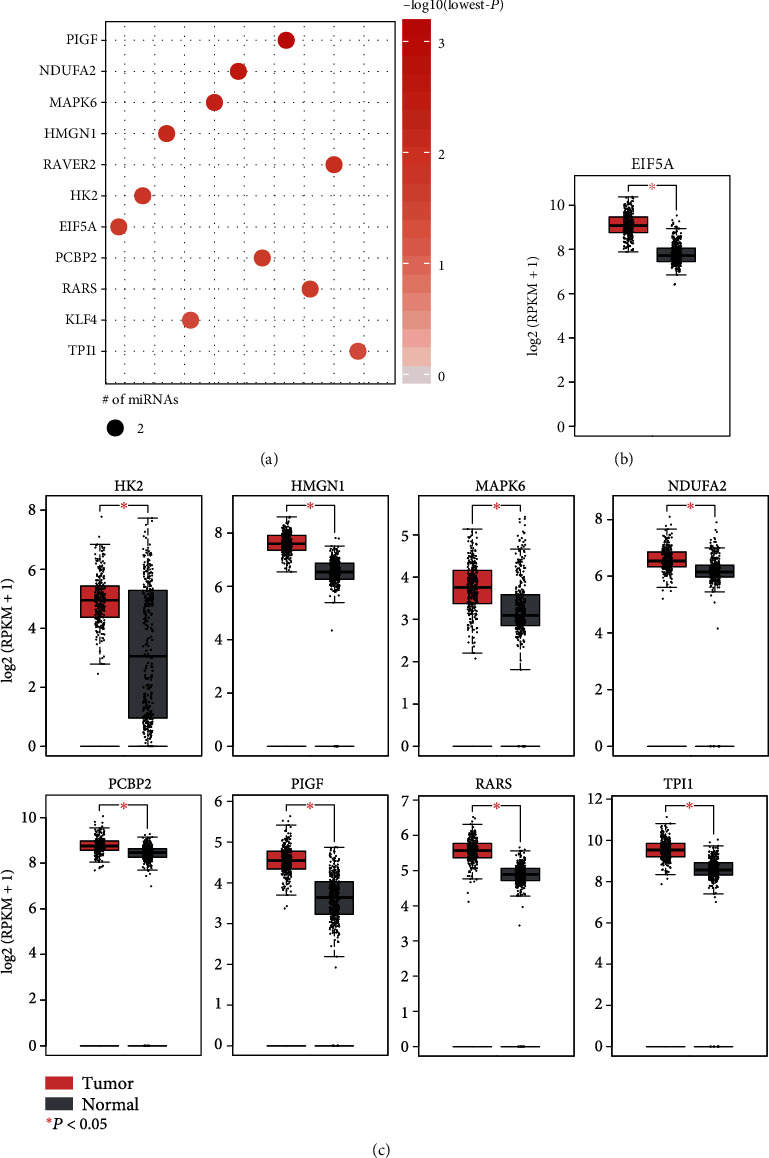
The target genes enriched by the tumorigenic microRNAs. (a) The statistical significance of the target genes enriched by the tumorigenic microRNAs. The nine target genes significantly up- or downregulated in colon adenocarcinoma were present in (b) and (c).

In general, the microRNAs functioned by binding to the 3′ untranslated regions (3′-UTRs) of the protein-coding genes. To investigate the target genes of these tumorigenic microRNAs, we first conducted ORA of these microRNAs, and then used the Pearson correlation coefficients (PCC) to refine these microRNA-target pairs. Given that *P* value < 0.001 and PCC < −0.15, we identified 11 genes, including PIGF, TPI1, KLF4, RARS, PCBP2, EIF5A, HK2, RAVER2, HMGN1, MAPK6, and NDUFA2, that were not only enriched by but also negatively correlated with these dysregulated microRNAs (Figure 3(a)). It should be noted that the correlation analysis was conducted on the gene and microRNA expression datasets of the TCGA COAD cohort. Moreover, these target genes were observed to be significantly upregulated in colorectal cancer (*P* < 0.05, Figures 3(b) and 3(c)) using the online tool GEPIA [15] (http://gepia.cancer-pku.cn/). Notably, in accordance with the GO terms by ORA, such as activation of MAPK activity and MAPK cascade, the MAPK6 was also enriched by the microRNAs, suggesting that the MAPK activity and cascade were enhanced by downregulating the microRNAs targeting MAPK6.

### 3.4. The Expression Patterns of the Tumorigenic microRNAs in Colorectal Adenocarcinoma

**Figure 4:**
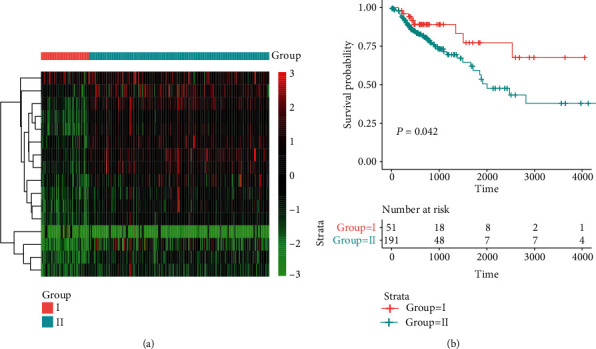
The expression patterns of the tumorigenic microRNAs in colon adenocarcinoma. (a) The expression profiles of the tumorigenic microRNAs in colon adenocarcinoma. (b) The survival analysis of the two groups stratified by the expression patterns of the tumorigenic microRNAs.

To further explore the role of the tumorigenic microRNAs in colorectal cancer, we also investigated their expression in the TCGA colon adenocarcinoma (COAD) cohort. Consistently, most of these microRNAs were also dysregulated in colon adenocarcinoma (*P* value < 0.05). However, some of the colon adenocarcinoma samples exhibited different expression patterns from the others. As shown in Figure 4(a), the samples could be stratified into two groups, I and II, based on multiple criteria calculated by R package *NbClust*. To analyze the association between this stratification and prognosis, the survival analysis revealed that the two groups showed significantly different overall survival, with longer overall survival in group I (Figure 4(b)). Interestingly, the samples with better prognosis had lower expression of these downregulated microRNAs than those with worse prognosis (Figures 4(a) and 4(b)), suggesting that there might be some other factors promoting the progression of colorectal cancer.

### 3.5. The Clinical Significance of the Tumorigenic microRNAs in CRC

**Figure 5:**
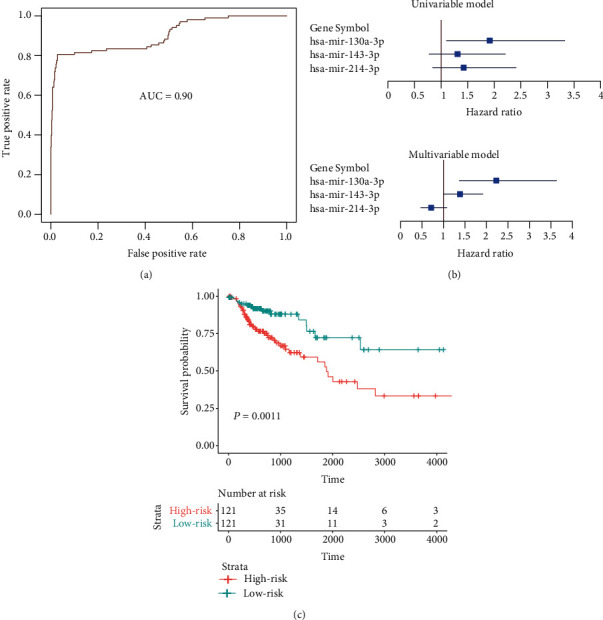
The clinical significance of the tumorigenic microRNAs in colon cancer. (a) The ROC curve for the adenoma and normal tissue prediction. (b) The statistical significance of the three microRNAs included in the multivariable Cox model. (c) The KM curve for the samples in high (red) and low risk (green) groups.

To further investigate the clinical significance of the tumorigenic microRNAs in CRC, we built machine learning and survival models to evaluate the discriminating ability between the CRC and normal tissues, and the prognostic value in CRC. The support vector machine (SVM) was trained based on the 20 tumorigenic microRNAs. The Receiver Operating Characteristic (ROC) curve showed that the area under the curve (AUC) value was about 90% (Figure 5(a)), suggesting that the tumorigenic microRNAs were capable of accurately discriminating the CRC from normal tissues. Moreover, a multivariable Cox regression model was conducted on the tumorigenic microRNAs, and the stepwise method was applied to selecting a combination of these markers. Specifically, three microRNAs, including hsa-mir-130a-3p, hsa-mir-143-3p, and hsa-mir-214-3p, were selected. Although hsa-mir-143-3p and hsa-mir-214-3p showed less significance in the univariable Cox model, combined with hsa-mir-130a-3p, the two microRNAs exhibited higher significance in the multivariable model (Figure 5(b)). Consistently, the Kaplan-Meier curve showed that the two risk groups based on the three microRNAs showed significantly different overall survival (Figure 5(c)).

## 4. Discussion

Noncoding RNAs, especially the microRNAs, have been found to play key roles in CRC development in intestinal tissue [16–19]. In this study, we identified 20 miRNA biomarkers dysregulated in the colon adenomas, among which, the mir-224-5p and mir-203a were upregulated. Consistently, mir-224-5p and mir-203a have been reported to be correlated with poor OS of CRC patients [20] and promote colorectal cancer proliferation and migration by targeting PDE4D [21], respectively. The functional enrichment analysis showed that MAPK activity and MAPK cascade were highly enriched by these tumorigenic microRNAs. Notably, in accordance with these GO terms, the MAPK6, targeted by the tumorigenic microRNAs, was also enriched by the microRNAs, suggesting that the MAPK activity and cascade were enhanced by downregulating the microRNAs targeting MAPK6. The correlation of the ERK/MAPK signaling pathway with proliferation and apoptosis of colon cancer cells has been reported previously [22]. The KEGG pathways were mainly enriched in the interactions between cell and extracellular matrix, such as endocytosis and adherens junction (Figure 3(b)), indicating that these interactions had been changed in adenoma. The endocytosis and adherens junction have been widely reported to be associated with tumorigenesis and progression in cancers [23, 24].

Furthermore, we also investigated the target genes of the tumorigenic microRNAs. Eleven genes, including PIGF, TPI1, KLF4, RARS, PCBP2, EIF5A, HK2, RAVER2, HMGN1, MAPK6, and NDUFA2, were identified to be frequently targeted by the tumorigenic microRNAs. Notably, the clinical and molecular significance of the target genes, such as PIGF [25], KLF4 [26], EIF5A [27], HK2 [28], HMGN1 [29], and MAPK6 [30], have been widely reported by previous studies.

In addition, we explored the role of the tumorigenic microRNAs in colorectal cancer using the datasets of the TCGA colon adenocarcinoma (COAD) cohort. Consistently, most of these microRNAs were also dysregulated in colon adenocarcinoma (*P* value < 0.05). The samples could be stratified into two groups, I and II, which showed significantly different overall survival, with longer overall survival in group I (Figure 4(b)). Interestingly, the samples with better prognosis had lower expression of these downregulated microRNAs than those with worse prognosis (Figures 4(a) and 4(b)), suggesting that there might be some other factors promoting the progression of colorectal cancer. Computationally, we built the SVM model and multivariable Cox regression model to evaluate the performance of tumorigenic microRNAs in discriminating the adenomas from normal tissues and risk prediction. The high AUC value and distinct overall survival rates between the two risk groups suggested that these tumorigenic microRNAs had the potential of diagnostic and prognostic value in CRC.

In summary, the present study revealed possible mechanisms and pathways that may contribute to the tumorigenesis of CRC, which could not only be used as CRC early detection biomarkers but also be useful for tumorigenesis mechanism studies.

## Data Availability

The data related to this manuscript can be found in GSE115513.
